# *De Novo* Transcriptome Assembly of Pummelo and Molecular Marker Development

**DOI:** 10.1371/journal.pone.0120615

**Published:** 2015-03-23

**Authors:** Mei Liang, Xiaoming Yang, Hang Li, Shiying Su, Hualin Yi, Lijun Chai, Xiuxin Deng

**Affiliations:** Key Laboratory of Horticultural Plant Biology, Ministry of Education, Key Laboratory of Horticultural Crop Biology and Genetic improvement (Central Region), MOA, Huazhong Agricultural University, Wuhan, Hubei, 430070, China; USDA-ARS-SRRC, UNITED STATES

## Abstract

Pummelo (*Citrus grandis*) is an important fruit crop worldwide because of its nutritional value. To accelerate the pummelo breeding program, it is essential to obtain extensive genetic information and develop relative molecular markers. Here, we obtained a 12-Gb transcriptome dataset of pummelo through a mixture of RNA from seven tissues using Illumina pair-end sequencing, assembled into 57,212 unigenes with an average length of 1010 bp. The annotation and classification results showed that a total of 39,584 unigenes had similar hits to the known proteins of four public databases, and 31,501 were classified into 55 Gene Ontology (GO) functional sub-categories. The search for putative molecular markers among 57,212 unigenes identified 10,276 simple sequence repeats (SSRs) and 64,720 single nucleotide polymorphisms (SNPs). High-quality primers of 1174 SSR loci were designed, of which 88.16% were localized to nine chromosomes of sweet orange. Of 100 SSR primers that were randomly selected for testing, 87 successfully amplified clear banding patterns. Of these primers, 29 with a mean PIC (polymorphic information content) value of 0.52 were effectively applied for phylogenetic analysis. Of the 20 SNP primers, 14 primers, including 54 potential SNPs, yielded target amplifications, and 46 loci were verified via Sanger sequencing. This new dataset will be a valuable resource for molecular biology studies of pummelo and provides reliable information regarding SNP and SSR marker development, thus expediting the breeding program of pummelo.

## Introduction

Pummelo (*Citrus grandis* (L.) Osbeck), also known as the female parent of sweet orange, is an important economical cultivated species and a valuable germplasm for citrus breeding. Pummelo is commercially cultivated in many countries, such as China, Thailand, Japan, Mexico and Israel, because pummelo fruits and juice have abundant antioxidant compounds, including vitamin C, carotenoids, flavonoids and limonoids, which can reduce the risk of oxidative-related diseases [1–4].

Although pummelo plays an important role in agricultural production and has beneficial health effects, little genomic and transcriptomic information is publicly available. As of January 2015, only 327 nucleotide and 58 EST sequences of pummelo were deposited in the GenBank database. Pummelo is an ancient species of the *Citrus* genus [5]; additionally, many accessions possess the mechanism of self-incompatibility [6]. Thus, it is difficult to sequence and assemble a whole genomic with a wide range of variability and high degree of heterozygosity. An alternative approach, namely transcriptome sequencing, which is used to generate massive expressed sequence tags (ESTs), was considered.

The transcriptome dataset is a valuable resource not only for expression profile construction but also for novel gene discovery [7–9] as well as for a large number of simple sequence repeat (SSR) and single nucleotide polymorphism (SNP) markers [10–12]. SSR and SNP molecular markers are very useful for variety identification, population structure analysis, and linkage map development and will contribute to the acceleration of the breeding program. In a previous study, 343 AFLPs and 335 SSRs were used to assess the genetic diversity of 110 pummelo germplasms [13]; thereafter, 178 pummelo genotypes were identified using 25 SNPs [14]. However, most of the existing markers were developed from other species in *Citrus*, mainly from *C.sinensis*, and few markers have been developed from pummelo.

Given that the number of the markers derived from pummelo is limited, Biswas et al. [15] first reported a set of pummelo EST sequences and developed corresponding markers through the traditional method of constructing a cDNA library, selecting clones and sequencing by the Sanger method. However, the throughput of the dataset was not sufficient to satisfy the needs of molecular biology and breeding studies. Furthermore, the traditional method was costly, time-consuming and cumbersome. Fortunately, next-generation sequencing (NGS) technologies, including the Roche (454) GS FLX, Illumina genome analyzer and Applied Biosystems SOLiD sequencer, are revolutionizing our ability to obtain massive transcriptome data [16]. Among these high-throughput sequencing technologies, the Illumina platform is the most widely used and is suitable for the discovery of variants and genes because it can produce large volumes of high-quality reads [17]. Moreover, with the support of paired-end sequencing and advanced assemblies, Illumina can be successfully applied to non-model organisms rather than only for model organisms [18–21].

‘Shatian’ pummelo is a representative pummelo cultivar with the characteristic of self-incompatibility [22]. In this study, we characterized the transcriptome information of ‘Shatian’ pummelo using the Illumina platform and developed SSR and SNP markers of pummelo. To our knowledge, this is the first report of a pummelo transcriptome used to construct a gene expression dataset with 12 G data. Such data provide an important resource for novel gene discovery and marker development and lay a solid foundation for future breeding efforts and molecular biology research on pummelo. In addition, we designed corresponding SSR and SNP primer pairs to test the accuracy of the *in silico* loci and obtained the diversity analysis of 44 citrus and relative accessions through SSR markers.

## Results

### Illumina Sequencing and *de novo* ‘Shatian’ pummelo transcriptome assembly

**Fig 1:**
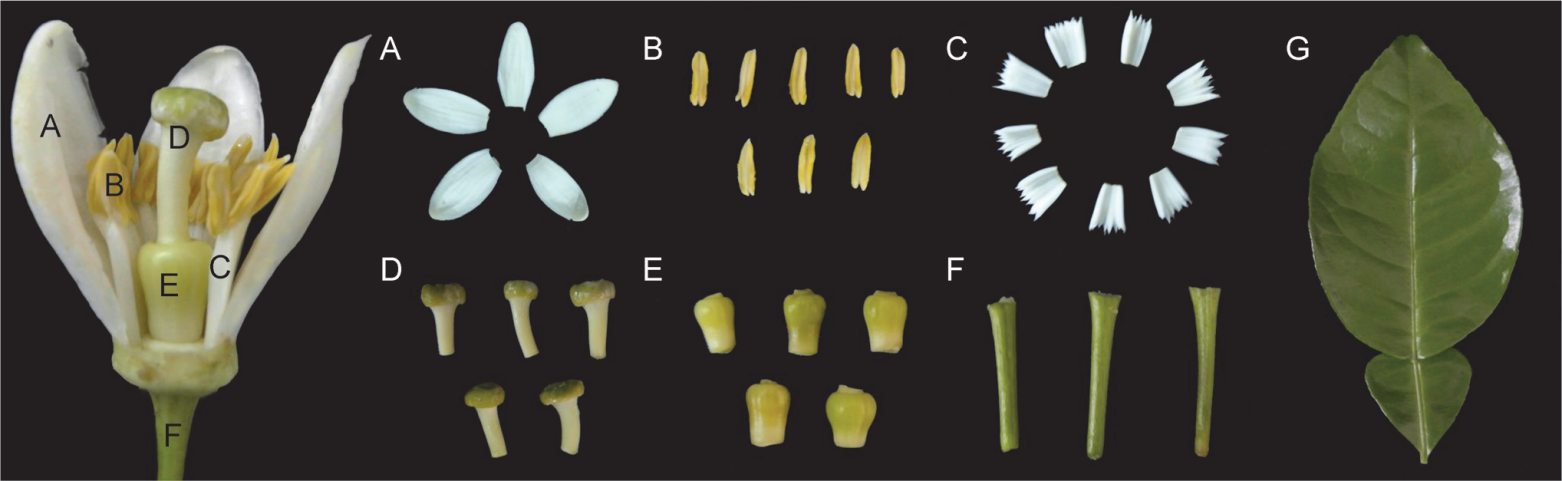
Six floral organs and young leaves of ‘Shatian’ pummelo that were used for library construction. (A) Petal. (B) Anther. (C) Filament. (D) Style. (E) Ovary. (F) Pedicel. (G) Leave. All of the floral organs were collected separately 10 days before the anthesis stage of the flowers.

To provide a comprehensive transcriptome platform for pummelo, we constructed a cDNA library of ‘Shatian’ pummelo through a mixture of RNA from seven sampled tissues (Fig. 1), i.e., petal, anther, filament, style, ovary, pedicel and leaf, and this library was named ‘Cg’ in this work and was sequenced using Illumina paired-end technology. The sequencing feature yielded approximately 149 million raw reads. After filtering out ambiguous, low-quality reads and reads with adaptors, the remaining 135,191,154 reads, encompassing 12,167,203,860 total nucleotides, were used for assembly. Trinity, a program for RNA-Seq transcriptome assembly without a reference genome [23], was used for our assembly, resulting in 101,235 contigs that contained 44 Mb sequences with an average length of 440 bp (Table 1). Of these contigs, 68.60% were shorter than 300 bp, 19.39% ranged from 300 to 1000 bp, and the remaining 12.01% were longer than 1000 bp (S1A Fig.).

**Table 1:** Summary of output sequences and assembly quality for ‘Shatian’ pummelo library.

	clean read	contig	unigene
Total Number	135,191,154	101,235	57,212
Total Length (bp)	12,167,203,860	44,540,712	57,770,072
Mean Length (bp)	90	440	1010
N50	-	1090	1630
Distinct Clusters	-	-	21,454
Distinct Singletons	-	-	35,758

Then, the contigs were further clustered and constructed into de Bruijn graphs. Each de Bruijn graph was processed independently to extract full-length splicing isoforms, namely, unigenes. Following these steps, we obtained 57,212 unigenes, of which distinct clusters and distinct singletons composed 37.5% and 62.5%, respectively. The mean size of the unigenes was 1010 bp, and 50% of the unigenes (N50) were 1630bp or longer (Table 1). The length distribution of the unigenes indicates that the assembled unigenes with lengths varying from 200 to 300 bp, 300 to 1000 bp and above 1000 bp accounted for 22.41%, 40.69% and 36.90% of the total, respectively (S1B Fig.).

### Annotation of the unigenes

To annotate 57,212 unigenes, a sequence similarity search based on the BLASTx algorithm was conducted against four public databases (i.e., the NCBI non-redundant (Nr) database, Swiss-Prot protein database, Clusters of Orthologous Groups (COG) database, and Kyoto Encyclopedia of Genes and Genomes (KEGG) database) with an E-value threshold of 10^–5^. In total, 39,584 unigenes were annotated to at least one of the mentioned databases, and 11,987 were matched with all of the databases (S2A Fig.). EST Scan was used to determine the sequence direction of the remaining 30.81% unigenes that were unmatched to any databases. Altogether, the directions of 40,497 unigenes were confirmed through the protein databases or ESTScan software.

**Fig 2:**
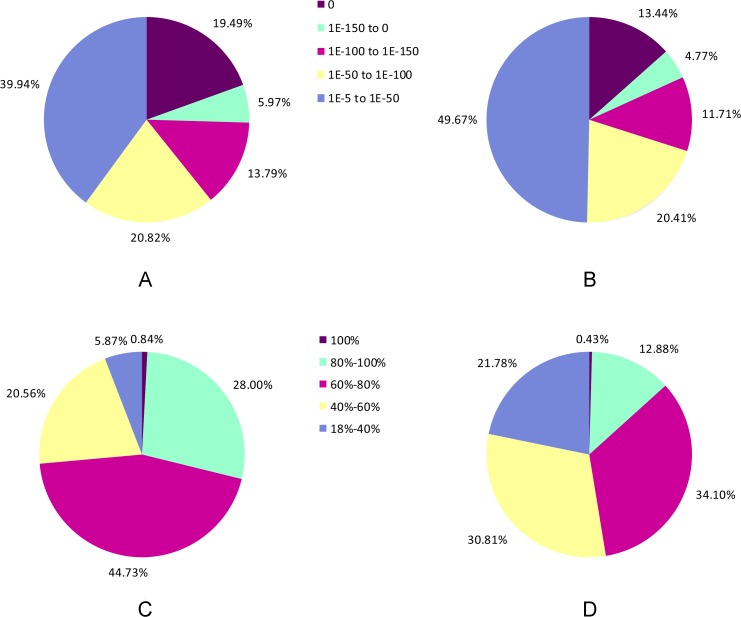
Characteristics of homology search for unigenes against Nr and Swiss-Prot protein databases. (A) E-value distribution of BLASTx hits for the ‘Shatian’ pummelo unigenes with an E-Value ≤ 1.0E-5 in the Nr database. (B)E-value distribution of BLASTx hits for the ‘Shatian’ pummelo unigenes with an E-Value ≤ 1.0E-5 in the Swiss-Prot database. (C) Similarity distribution of the top BLASTx hits for the ‘Shatian’ pummelo unigenes with an E-Value ≤ 1.0E-5 in the Nr database. (D) Similarity distribution of the top BLASTx hits for the ‘Shatian’ pummelo unigenes with an E-Value ≤ 1.0E-5 in the Swiss-Prot database.

Of the 57,212 unigenes, approximately 70% (39,488) were aligned with known proteins in the Nr database, where as approximately 46% (26,100) were annotated to the Swiss-Prot database. Among these annotated unigenes, 60.06% of Nr mapped, and 50.33% of Swiss-Prot hits had very strong homology, with an E-value≤1.0E-5 (Fig. 2A and 2B). The top hits with a similarity greater than 80% against the Nr and Swiss-Prot databases accounted for 28.84% and 13.31%, respectively (Fig. 2C and 2D). From the Nr results, we found that 23.91% of the unigenes were closely related to *Vitis vinifera*, followed by *Ricinus communis* (21.33%), *Populus balsamifera subsp*. *trichocarpa* (17.52%) and *Amygdalus persica* (14.26%) (S2B Fig.).

### Function classification of the unigenes

**Fig 3:**
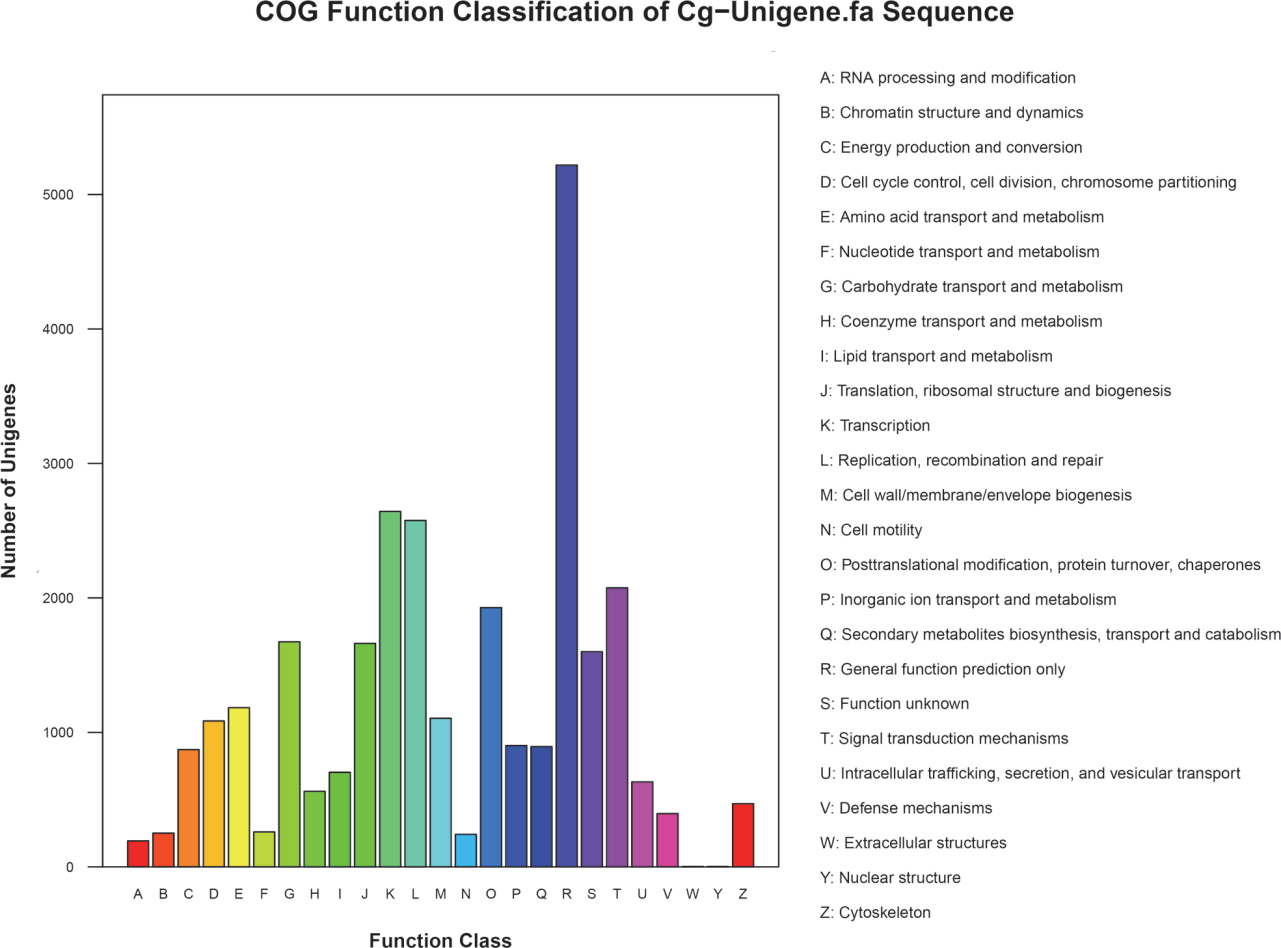
Clusters of orthologous group (COG) classification of ‘Shatian’ pummelo assembled unigenes. Out of 57,212 unigenes, 15,317 were grouped into 25 COG functional clusters, and the cluster of general function prediction represented the largest group, accounting for 17.91%.

The COG database, whose protein sequences are encoded in complete genomes, including bacteria, algae and eukaryotes [24], was used to functionally classify the data. Of the 57,212 unigenes, 15,317 (26.77%) were categorized into 25 functional clusters (Fig. 3). Because some of the unigenes were annotated into more than one classification, we obtained 29,134 functional terms. Among these classifications, the cluster for general function prediction only (5219; 17.91%) represented the largest group, followed by the clusters for transcription (2643; 9.07%); replication, recombination and repair (2576; 8.84%); signal transduction mechanisms (2075; 7.12%); posttranslational modification, protein turnover, and chaperones (1928; 6.62%); carbohydrate transport and metabolism (1674; 5.75%); translation, ribosomal structure and biogenesis (1662; 5.70%); and function unknown (1600; 5.49%). The percentages of the other categories were less than 5% (Fig. 3).

**Fig 4:**
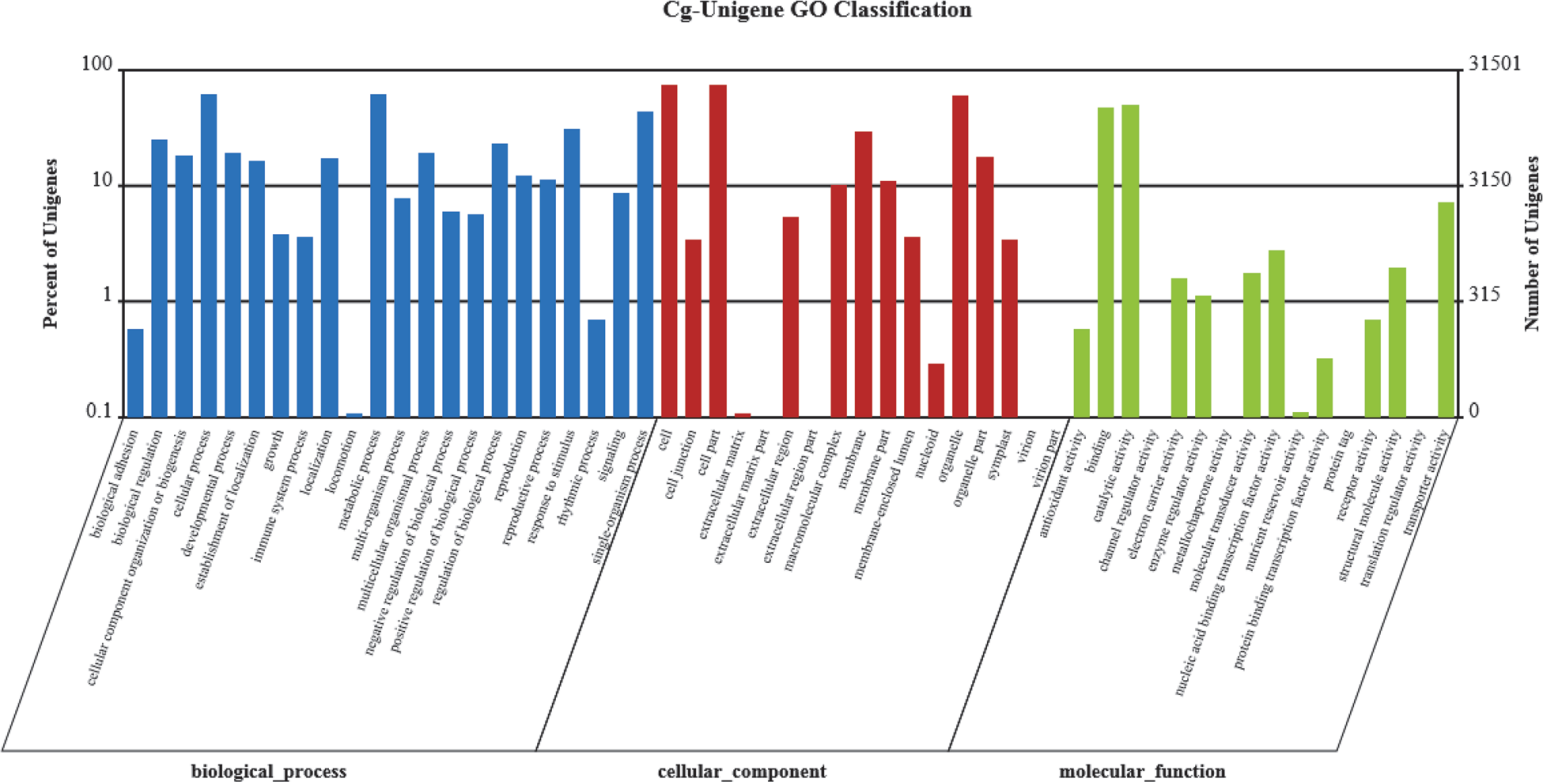
Gene ontology (GO) classification of ‘Shatian’ pummelo assembled unigenes. A total of 31,501 unigenes with BLASTx matches were assigned to three main categories: biological processes (125,432; 49.14%), cellular components (93,102; 36.48%), and molecular functions (36,709; 14.38%).

To further classify the functions of ‘Shatian’ pummelo unigenes, 39,488 unigenes with Nr annotation were submitted to the GO database consisting of molecular function, cellular component and biological process. A total of 31,501 unigenes were classified into 55 sub-categories with 255,243 functional terms (Fig. 4). Nearly half of the assignments were classified into biological process, and 36.48% and 14.38% fell into cellular component and molecular function, respectively. Under the biological process category, cellular process (19,618; 15.64%), metabolic process (19,292; 15.38%) and single-organism process (13,850; 11.04%) represented the major classifications. Furthermore, 2715 unigenes were assigned to the signaling category. Within the cellular component category, 23,617 unigenes were concentrated in the groups of the cell and the same number of unigenes in the groups of the cell parts. Interestingly, 19,117 unigenes were involved in the group of components of organelles. In the molecular function category, catalytic activity (15,976; 43.52%) and binding (15,027; 40.94%) were the most abundant sub-categories, whereas the other groups accounted for a percentage of less than 10% (Fig. 4).

To better understand the specific network of these transcripts, we annotated the 57,212 unigenes using the KEGG [24] database. As the results showed, 23,219 unigenes were mapped into 5 main categories and 128 KEGG pathways. As expected, most of the unigenes (19,780; 85.19%) fell into the metabolism category. In this category, global map, carbohydrate metabolism, lipid metabolism, and biosynthesis of other secondary metabolites were the most represented subcategories.

Genetic information processing, involving translation, folding, sorting and degradation, replication and repair, and transcription, contained 25.88% of the assembled unigenes. In addition, 8.95%, 7.63% and 4.94% of the unigenes were aligned to organismal systems, environmental information processing and cellular processes, respectively (S1 Table).

In short, the results of the functional analysis revealed the feasibility of obtaining transcriptome sequences through high-throughput technology, even for non-model organisms with complex genomes. The transcriptome is an important resource for molecular marker development, and further experiments on marker mining are necessary to illustrate the value and to boost the availability of the sequence dataset.

### SSR mining and primer design

SSR markers are characterized by co-dominance and are highly polymorphic, and they are therefore useful in the field of genetic mapping and diversity analysis. Using the MISA tool, we identified 10,276 potential SSRs distributed in 8405 sequences, of which 1,477 unigenes contained multiple SSR loci. The distribution of all of the SSR loci is shown in Table 2. Tri-nucleotide repeats accounted for 30.41%, closely followed by dinucleotide motifs (26.06%), whereas tetra-, penta-, and hexa-nucleotide repeats represented only approximately 2%. Altogether, 192 repeat types of nucleotide motifs were detected. The AG/CT (1674, 16.29%) type was the most common, excluding repeat types of mononucleotide motifs.

**Table 2:** Summary of the distribution of identified SSRs and the major type of each motif.

SSR motif	Number of repeat units								Total (%)	Major repeat type (%)
	4	5	6	7	8	9	10	>10		
Di-nucleotide repeat	0	0	832	569	429	356	304	188	2678 (26.06%)	AG/CT (62.51%)
Tri-nucleotide repeat	0	1583	868	617	57	0	0	0	3125 (30.41%)	AAG/CTT (27.49%)
Tetra-nucleotide repeat	0	190	39	0	0	0	0	0	229 (2.23%)	AAAG/CTTT (27.07%)
Penta-nucleotide repeat	225	20	0	0	0	0	0	0	245 (2.38%)	AAAAT/ATTTT (33.06%)
Hexa-nucleotide repeat	285	0	0	0	0	0	0	0	285 (2.77%)	AAAAAT/ATTTTT (6.32%)

**Fig 5:**
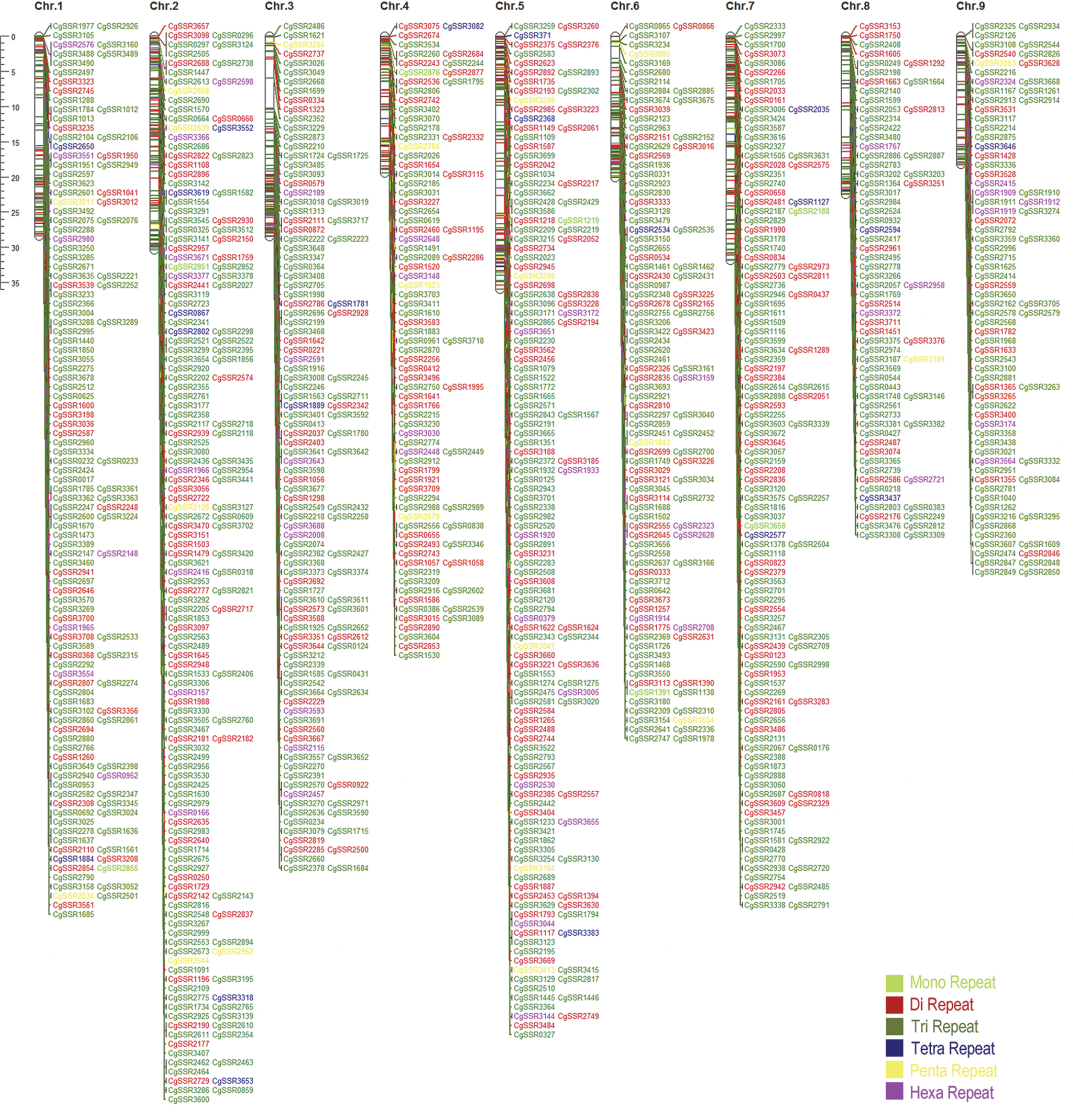
Chromosome maps of 1035 SSR loci with high-quality primers along the nine chromosomes of *C.sinensis*.

To provide convenient access for ‘Shatian’ pummelo molecular markers, we designed five primers for each potential SSR locus and subsequently eliminated low-quality primers. A total of 1174 SSR loci with high-quality primers were obtained (S2 Table). The distribution of these 1174 SSRs on the nine chromosomes of *C.sinensis* was analyzed using local BLASTn with a cut-off of 1.0E-5. The results indicate that 88.16% (1035) of the SSR loci were localized, presenting a relatively uniform distribution on nine chromosomes. Chromosome 2, containing 165 loci, represented the highest density (5.35 SSR/Mb), whereas chromosome 8 was the lowest with 72 loci (3.17 SSR/Mb) (Table 3). Furthermore, based on the exact positions of the localized genes on the chromosomes, we drew the chromosome map of the 1035 SSR loci using the Mapchart software (Fig. 5).

**Table 3:** Summary of the chromosome distribution of 1035 SSR loci in the *C.sinensis* genome.

Chromosome	No. of localization	Percentage (%)	Size of chromosome	Density (SSR/Mb)
chr.1	132	11.24	28.8	4.58
chr.2	165	14.05	30.84	5.35
chr.3	122	10.39	28.71	4.25
chr.4	88	7.50	19.95	4.41
chr.5	145	12.35	36.15	4.01
chr.6	109	9.28	21.18	5.15
chr.7	121	10.31	32.21	3.76
chr.8	72	6.13	22.71	3.17
chr.9	81	6.90	18.45	4.39
Total	1035	88.16	239	4.33

### SSR marker polymorphism

A total of 100 primer pairs were randomly selected and synthesized for wet-lab validation, and 87 primers successfully produced clear banding patterns upon polyacrylamide gel electrophoresis, of which 82 were of the expected sizes, and the remainder was larger than the expected sizes. Unsurprisingly, the observed sizes of some of the primers were larger than expected because the PCR template was genomic DNA, whereas the primers were designed according to cDNA.

To evaluate the utility of the SSR markers for phylogenetic analysis, 29 SSR primers selected from 82 primer pairs with desirable sizes were used to evaluate the genetic relationship among 44 citrus and relative accessions. The average amplified allele number of 29 SSR primers was 5.4, with a maximum of 10 and a minimum of 2 (Table 4). The polymorphic information content (PIC) values ranged from 0.35 to 0.79 with a mean value of 0.52 (Table 4). The observed heterozygosity (*Ho*) varied from 0.41 to 0.80, whereas the expected heterozygosity (*He*) varied from 0.11 to 0.80. The mean *Ho* and *He* values were 0.54 and 0.30, respectively.

**Table 4:** Characteristics of 29 primer pairs possessing polymorphic SSR loci as tested in 44 different citrus accessions and relatives.

Primer name	Motif	No. of allele	Ho	He	PIC
CgSSR0865-3	(CAT)6	4	0.5733	0.1364	0.5173
CgSSR0867-2	(ATTA)5	5	0.4548	0.3636	0.4209
CgSSR1023-3	(AAAAT)4	6	0.7348	0.1136	0.6948
CgSSR1057-2	(AG)7	4	0.478	0.1818	0.4374
CgSSR1261-4	(CTC)8	7	0.7603	0.4773	0.7218
CgSSR1308-2	(AGGAA)4	6	0.5537	0.2045	0.4542
CgSSR1630-1	(CTT)5	6	0.4982	0.2955	0.4632
CgSSR1663-1	(CT)7	5	0.5744	0.1591	0.5192
CgSSR1714-1	(CAT)6	4	0.4928	0.1364	0.4097
CgSSR1884-2	(TAAA)5	8	0.5457	0.3182	0.516
CgSSR1909-2	(AAACCA)4	4	0.5808	0.7955	0.4959
CgSSR2034-3	(AAATA)4	5	0.5664	0.2955	0.5316
CgSSR2189-2	(CGCCGA)4	3	0.509	0.4545	0.4097
CgSSR2380-2	(AAAAT)4	6	0.3794	0.3182	0.3512
CgSSR2505-2	(CTT)7	4	0.4336	0.3409	0.3782
CgSSR2511-2	(CT)8	10	0.8066	0.5909	0.7858
CgSSR2591-1	(CCCGTC)4	4	0.571	0.2045	0.4788
CgSSR2639-2	(GGCTT)5	7	0.7474	0.6136	0.7096
CgSSR2746-1	(ATAAA)4	6	0.5628	0.2727	0.5359
CgSSR2784-1	(GATCC)4	6	0.4858	0.3182	0.4397
CgSSR2802-3	(CAGG)5	5	0.6402	0.2045	0.5823
CgSSR3041-3	(GTCGT)4	6	0.5718	0.3409	0.5233
CgSSR3053-4	(TTTAG)4	4	0.4223	0.3182	0.3704
CgSSR3164-3	(TAAAT)4	5	0.6074	0.2727	0.5599
CgSSR3318-4	(GCTC)5	4	0.5752	0.3864	0.5283
CgSSR3383-4	(TGAC)6	6	0.7482	0.3409	0.7111
CgSSR3407-1	(AAG)6	5	0.586	0.0682	0.5114
CgSSR3619-3	(ATTT)5	7	0.7549	0.25	0.7145
CgSSR3653-4	(GTTA)5	6	0.413	0.2273	0.3852
Mean		5	0.5742	0.3000	0.5224

Note: Ho: Observed heterozygosity; He: Expected heterozygosity; PIC: Polymorphism information content. The corresponding detailed information for the 29 primers is displayed in S4 Table.

**Fig 6:**
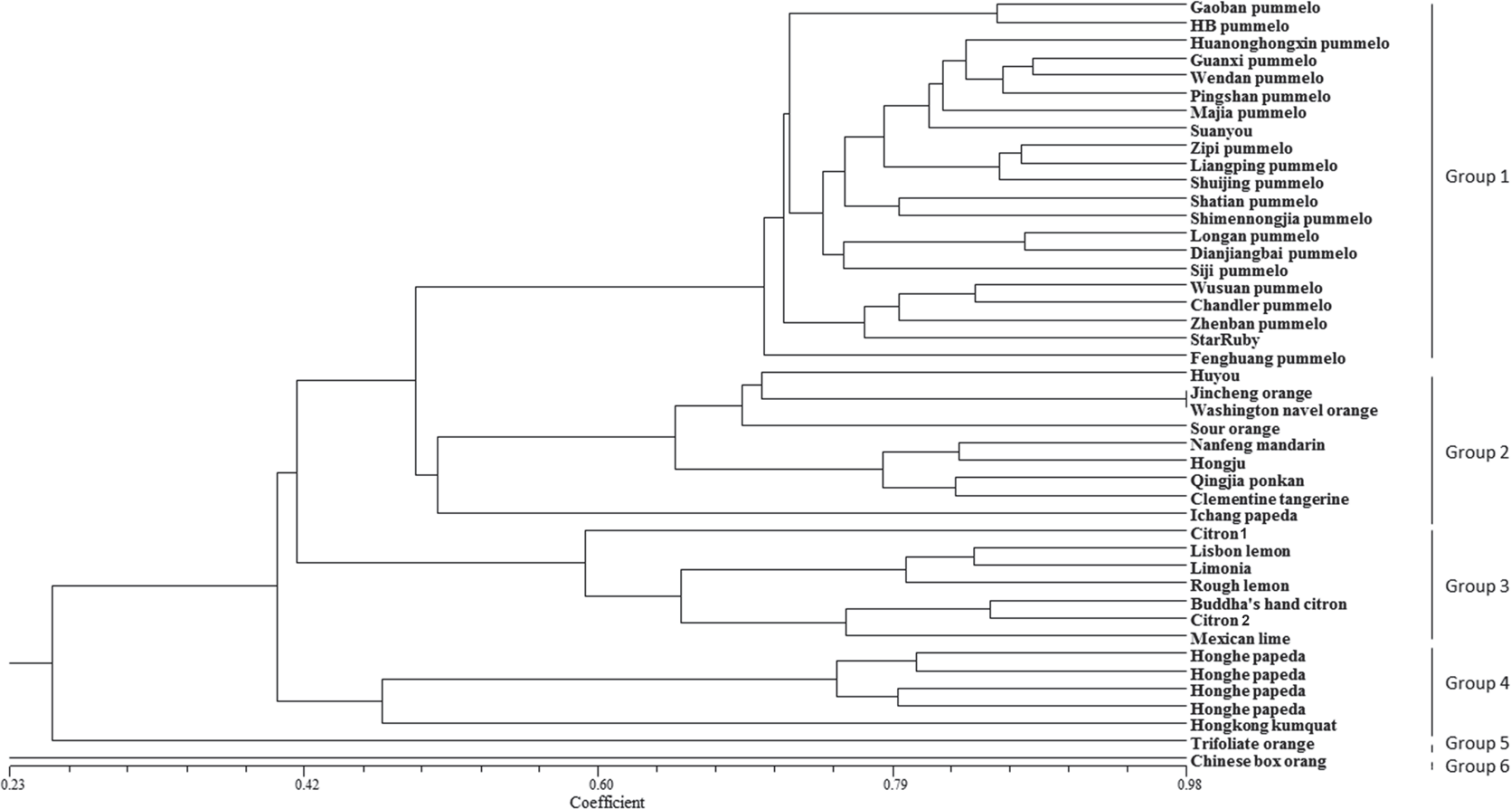
Phylogenetic relationships between 44 citrus and relatives based on 29 SSR loci. All of the accessions were classified into six distinct genetic groups at the Dice’s coefficient of approximately 0.23.

We then calculated the similarity matrix of 158 alleles of 29 polymorphic SSR markers according to Dice’s coefficient and clustered the genotypes based on the UPGMA method. As shown in Fig. 6, all of the studied genotypes were clearly different and were classified into six distinct genetic groups with a Dice’s coefficient of approximately 0.23. Then, 21 members of *C*. *grandis* were grouped into a single distance cluster. *C*. *paradise*, *C*. *sinensis*, *C*. *aurantium* and *C*. *ichangensis* were grouped together with *C*. *reticulata*, whereas *C*. *aurantifolia*, *C.limon*, *C*. *medica* and *C*. *jambhiri* were placed in the same cluster. *F*. *hindsii* and *C*. *hongheensis* were clustered together. *P*. *trifoliata* had a considerably distant relationship to any *Citrus* species, and *Severinia buxifolia*, as an out group, was placed in a single distinct cluster.

### SNP mining and validation

**Fig 7:**
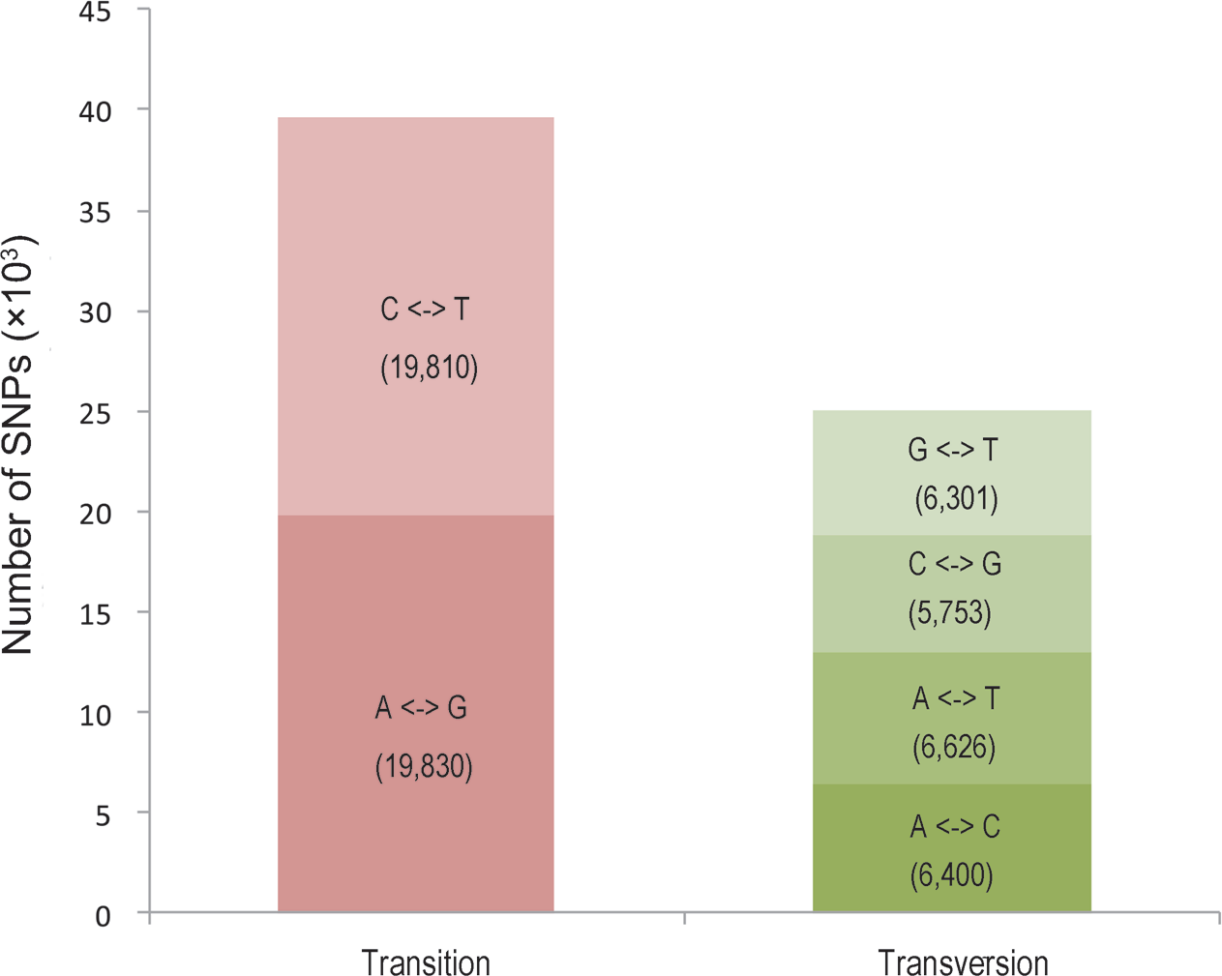
Frequency distribution of the putative SNPs based on the variation types. A total of 64,720 putative SNP loci were found in 19,172 unigenes, and the transition and transversion types accounted for 61% and 39%, respectively.

SNP molecular markers are powerful tools for gene mapping and molecular marker-assisted selection because they have the most abundant variations in genomes. In this study, 64,720 putative SNP loci were identified within 19,172 unigenes, with 62.01% unigenes including two or more SNPs. Of the 64,720 SNPs, 61.24% belonged to transition type (C/T and A/G), and 38.75% were transversion type (G/T, C/G, A/T and A/C) (Fig. 7). There were almost as many C/T types (19,810) as A/G types (19,830) in the transition types, whereas within the transversion types, A/T type (6,626) was the most common variation, followed by types A/C (6,400), G/T (6,301), and C/G (5,753).

The genomic DNA of ‘Shatian’ pummelo was used as a template to validate the accuracy of the putative SNPs. A total of 20 SNP primer pairs were designed, and 14 primers presumptively including 53 potential SNP loci yielded target amplifications (S4 Table; S5 Table). The results of sequencing these 14 primers by the Sanger method indicated that 48 SNPs (90.57%) whose quality scores varied from 23 to 99 were validated (S5 Table).

## Discussion

### Illumina paired-end sequencing and assembly

NGS technology, which is characterized by unprecedented high throughput, convenience and cost-effectiveness, is likely to become the method of choice for transcriptome sequencing, which is an important molecular biology tool that is widely used in various studies, such as in generating different dynamic views of gene expression [25–27], the discovery of novel genes or transcripts [28,29], splicing activity [30], and molecular marker mining [31–34]. The Illumina platform, as a commercially available NGS technology, was initially used for organisms with reference genomes due to the limitation of short read lengths and the available assembly software [35,36]. In recent years, with the introduction of advanced algorithms, massive short reads have been successfully assembled *de novo* even without reference sequences [23,37,38]. In addition, the latest paired-end sequencing technology has further increased the sequencing depth and expanded the assembly length [39].

In this study, a transcriptome dataset of the pooled RNA from seven ‘Shatian’ pummelo tissues was generated using Illumina paired-end sequencing. More than 135 million 90-bp paired-end reads were produced, encompassing 12 Gb of sequence data. The assembly results show that a feasible strategy could be used to assemble a large number of Illumina short reads of non-model woody plants. All of the short reads were assembled into 57,212 unigenes with a mean length of 1010 bp, which was longer than in many other studies of woody plants [40–43]. The N50 of the unigenes was 1630 bp in our study, whereas Du F et al. [44] obtained 49,991 unigenes with an N50 of 988bp using a similar method. Previous publications [45] indicated that an accurate and effective assembly tends to have a longer mean length and a larger N50 value.

### Annotation and Functional Classification of Unigenes

The lack of a reference genome limits the prediction of protein function and utilization of the transcriptome dataset. To overcome this problem, we submitted all of the unigenes to BLASTx analysis against four public databases (Nr, Swiss-Prot, COG and KEGG). As a result, 69.19% of the unigenes had homologous hits with the above protein databases, which was similar to the value for lily (72.20%) [44] but less than for Chinese bayberry (79.55%) [46] and radish (92.09%) [47]. These unigenes that were not annotated to any known proteins may be novel transcripts that are unique to pummelo; however, we cannot exclude the possibility of sequencing error. In our research, 39,488 (69.02%) and 26,100 (45.62%) unigenes had homologous hits with the Nr and Swiss-Prot databases, respectively. Consistent with previous studies [21,40,44], the majority of unigenes matched with the Swiss-Prot database, displaying a reliable annotation in the Nr database. Among all of the pummelo unigenes, CL2175.Contig1_Cg, Unigene24287_Cg, and Unigene16912_Cg were the top 3 most abundant transcripts and were uniquely mapped with approximately1.76, 0.98, and 0.45 million reads, respectively. Based on the BLASTx analysis, CL2175.Contig1_Cg and Unigene16912_Cg were separately aligned with certain proteins of *C.sinensis*, indicating that an accurate transcriptome dataset was obtained due to the confirmed genetic relationship between *C.grandis* and *C.sinensis* [48]. The Unigene24287_Cg, without any mapping to any protein database, was assumed to be a highly expressed transcript that is unique to pummelo.

Additionally, 15,317 unigenes were assigned to 25 COG clusters with 29,134 functional terms, and 31,501 unigenes were classified into 55 GO sub-categories with 255,243 functional terms. A total of 23,219 unigenes were mapped to 128 KEGG pathways, in which metabolism represented the richest pathway. Chao Liang et al. [49] identified the key genes involved in fatty acid and unsaturated fatty acid biosynthesis using *de novo* assembly for the transcriptome dataset. Based on the results of the annotation, we also found that some genes were annotated, such as S-like RNase, CL6572.Contig1_Cg, Unigene4758_Cg, CL89.Contig1_Cg, and Unigene9342_Cg, that share a similar sequence with S-RNase, regulating gametophytic self-incompatibility [50,51]. Altogether, the information of transcriptome sequences is valuable for the study of molecular mechanisms; meanwhile, the results demonstrate the effectiveness and reliability of Illumina paired-end sequencing.

### Markers identification and validation

EST sequences are importance resources for the development of markers, such as SSR and SNP. For *C.grandis*, the available SSR and SNP markers are insufficient, and prior to this work, only Biswas et al. and Chai et al. had published relevant studies, obtaining 115 SNPs and 212 SSRs from 2,228 unigenes via Sanger sequencing [15,52]. In our study, 10,276 potential SSRs and 64,720 candidate SNPs were identified from the sequences that were obtained by the NGS technology. Those SSRs were detected from 8405 sequences with a frequency of one SSR per 5.6 kb. This frequency was similar to the results for other citrus as reported by Chen et al. (1/5.2 kb) [53] and for wheat as observed by Peng et al. (1/5.46 kb) [54]. All of the SNP loci were distributed in 19,172 unigenes with an average of one SNP per 892 bp. The density was similar to salmon louse (1/768 bp) [55] and higher than for wheat (1/3.6 kb) [7] and red clover (1/1.5 kb) [56]. To check the redundancy of the 8405 sequences with SSR loci, a BLASTn alignment was conducted against the 212 pummelo EST sequences that were used for SSR mining by Biswas et al. and Chai et al. [15,52]. Only 2.82% of the sequences exhibited homology, indicating that our SSR set made a great contribution to SSR identification for *C.grandis*.

The primers of SNP and SSR were designed to test the validity of the *in silico* loci. Because SSRs have been extensively and effectively used in genetic analysis, we developed corresponding primers for 1174 SSR loci to provide easy access for researchers; additionally, 88.16% of the loci were localized uniformly to nine chromosomes of *C.sinensis*. Then, 100 SSR primers were randomly selected, of which 87 yielded successful bands, and 82 were of the expected size. The success rates of amplification were different among the various plants: 100% for peanut [57], 90% for sweet potato [21], 82% for alfalfa [58], and 72% for apricot [59]. Furthermore, of the 20 SNP primers, 14 successfully amplified target fragments, and more than 85% containing low- and high-quality loci were confirmed via Sanger sequencing, which was slightly higher than the 76% accuracy reported by Yu et al. [60]. In total, the results indicated that NGS technologies can be used to develop abundant SNP or SSR markers with high efficiency and accuracy.

### SSR marker genetic diversity analysis

SSR markers were subjected to genetic diversity analysis, and markers with at least 3 alleles were identified, which was better than the previous result by Chai et al. [52]. The PIC value in our study (0.52) was greater than 0.5, suggesting that the majority of SSR markers were at a high polymorphism level and were suitable for genetic diversity study. However, the PIC was lower than in Chai’s report (0.87) [52]. The possible reason for this result is that fewer markers were used, and the primers were selected randomly rather than intentionally selected for high polymorphism.

According to the hypothesis of Barrett and Rhodes [61], there are only three major ancestral citrus species, citron (*C.medica* L.), mandarin (*C.reticulata* Blanco) and pummelo (*C.grandis* Osb.). Other cultivated species were derived from hybridization between these major ancestral species and closely related genera [62]. Our findings support this hypothesis. From the dendrogram, we found that pummelo, mandarin, and citron were clearly distinguished from each other and were allied with three independent clusters. All of the pummelos were grouped together and formed a single cluster, excluding ‘huyou’ (*C.paradisi* Macf.). In our results, ‘huyou’ exhibited a close relationship with sweet orange and sour orange, confirming the hypothesis that ‘huyou’ is a hybrid between pummelo and sweet orange. Sweet orange, sour orange and mandarin all belong to the same cluster; therefore, our findings indicate that, as many researchers have assumed [48], mandarin has a significant genome contribution to these two types of orange. Lime, lemon, and limonia had close relationships with citron, indicating that citron might be a parent species [63–65]. Moreover, trifoliate orange, kumquat, and papeda were separated from citrus, although there were some similarities in the morphological characteristics and metabolic composition between citrus and trifoliate orange, kumquat, and papeda [66–68]. Taken together, these SSR markers that were developed based on the transcriptome dataset could elucidate a relatively unambiguous genetic relationship among Citrus and its relatives.

## Conclusion

This work is the first transcriptome study on the non-model plant *C.grandis* using the NGS technology, which provides a comprehensive available transcriptome dataset expressed in the floral organs and young leaves. In total, 57,212 unigenes were assembled, of which 39,584 could be annotated to known proteins of the public databases. Based on the dataset, we identified the top three most abundant transcripts and some S-like RNase genes that were related to the self-incompatibility mechanism. Moreover, a large number of SNPs and SSRs were identified, and high-quality primers of 1174 SSR loci were designed, 88.16% of which were localized to the chromosomes of sweet orange. Ultimately, a relatively unambiguous genetic relationship among citrus and its relatives was elucidated via 29 SSR markers that were developed from the transcriptome dataset. The enrichment results not only indicate that the assembled data were reliable and accurate and that massive short reads could be effectively assembled without reference sequences but also demonstrate that the transcriptome dataset could provide abundant genetic resources for novel gene identification and marker development.

## Methods

### Plant material and RNA and DNA extraction

‘Shatian’ pummelo was cultivated in the Guangxi Citrus Research Institute in Guilin City, Guangxi, China. The sampled tissues included young leaves and six floral organs (Fig. 1), i.e., petal, anther, filament, style, ovary, and pedicel, collected separately 10 days before the anthesis stage of the flowers. To examine the polymorphism of the detected SSR markers in our study, fresh young leaves of 41 citrus accessions and 3 related species (Table 5) were collected. All of the tissue and leaf samples were immediately frozen in liquid nitrogen and stored at −80°C until further extraction. The RNA of each tissue was isolated in terms of the protocol of Liu et al. [69], and the RNAs of the seven different tissues were equally pooled. The DNA was extracted from young leaves using the method described by Cheng et al. [70].

**Table 5:** Citrus genotypes and related species used in the study for SSR validation.

ID	Common name	Scientific name	ID	Common name	Scientific name
1	Gaoban pummelo	C. grandis (L.) Osbeck	23	Jincheng orange	C. sinensis (L.) Osbeck
2	Huanonghongxin pummelo	C. grandis (L.) Osbeck	24	Washington navel orange	C. sinensis (L.) Osbeck
3	Wusuan pummelo	C. grandis (L.) Osbeck	25	Sour orange	C. aurantium L.
4	Zipi pummelo	C. grandis (L.) Osbeck	26	Honghe papeda	C. hongheensis Y.L.D.L
5	Shatian pummelo	C. grandis (L.) Osbeck	27	Honghe papeda	C. hongheensis Y.L.D.L
6	Guanxi pummelo	C. grandis (L.) Osbeck	28	Honghe papeda	C. hongheensis Y.L.D.L
7	Pingshan pummelo	C. grandis (L.) Osbeck	29	Honghe papeda	C. hongheensis Y.L.D.L
8	Liangping pummelo	C. grandis (L.) Osbeck	30	Nanfeng mandarin	C. reticulata Blanco
9	Shuijing pummelo	C. grandis (L.) Osbeck	31	Hongju	C. reticulata Blanco
10	Wendan pummelo	C. grandis (L.) Osbeck	32	Qingjia ponkan	C. reticulata Blanco
11	Longan pummelo	C. grandis (L.) Osbeck	33	Clementine tangerine	C. reticulata Blanco
12	Dianjiangbai pummelo	C. grandis (L.) Osbeck	34	Ichang papeda	C. ichangensis Swing.
13	Chandler pummelo	C. grandis (L.) Osbeck	35	Buddha's hand citron	C. medica var. sarcodactylis
14	Fenghuang pummelo	C. grandis (L.) Osbeck	36	Citron1	C. medica L.
15	HB pummelo	C. grandis (L.) Osbeck	37	Citron2	C. medica L.
16	Majia pummelo	C. grandis (L.) Osbeck	38	Lisbon lemon	C. limon (L.) Burm.f.
17	Shimennongjia pummelo	C. grandis (L.) Osbeck	39	Limonia	C. limon(L.) Burm.f.
18	Suanyou	C. grandis (L.) Osbeck	40	Rough lemon	C. jambhiri Lush.
19	Zhenban pummelo	C. grandis (L.) Osbeck	41	Mexican lime	C. aurantifolia (Christ.) Swing.
20	Siji pummelo	C. grandis (L.) Osbeck	42	Trifoliate orange	P. trifoliata (L.) Raf.
21	Star Ruby	C. paradisi Macf.	43	Hongkong kumquat	F. hindsii Swing.
22	Huyou	C. paradisi Macf.	44	Chinese box orange	Severinia buxifolia (Poir.) Ten.

### Library construction and sequencing

The transcriptome sequencing of the constructed library was performed by the Beijing Genomics Institute (BGI, Shenzhen, China) according to the manufacturer’s instructions. Initially, RNA was treated with DNase I. Poly (A) mRNA was isolated from the treated RNA using magnetic oligo (dT) beads, and enriched mRNA was then broken into short fragments by mixing with the fragmentation buffer. Subsequently, first-strand and second-strand cDNA was successively synthesized using these fragments as templates. The double-stranded cDNA was resolved for end-repair, and a single adenine was added to the 3’ end. To distinguish between different samples, sequencing adapters were ligated to the short fragments, and suitable fragments of approximately 200 bp were selected for PCR amplification. An Agilent 2100 Bioanalyzer and an ABI StepOnePlus Real-Time PCR System were used to validate the quantification and qualification of the sample library. Finally, the cDNA library was sequenced using Illumina HiSeq 2000. The sequence data were deposited in the NCBI Sequence Read Archive (http://www.ncbi.nlm.nih.gov/Traces/sra) under accession number SRP051793.

### Data filtering and *de novo* assembly

To separate high-quality sequences from raw reads for further analysis, we conducted a filtering process that discarded reads containing adaptors or unknown nucleotides that were larger than 5% and low-quality reads with more than 20% of the bases of quality value ≤ 10.The short read assembly program Trinity [23] was used for *de novo* assembly of the clear reads through the following steps. First, clean reads with a certain length of overlap were extended to form longer contigs, and these contigs were clustered and constructed into individual de Bruijn graphs. Then, the reads were mapped back to the contigs for quality validation. Based on the outcome of the validation, the contigs with little mapping were removed, and the high-quality contigs were further assembled into unigenes. Finally, the obtained unigenes were divided into two classes, namely clusters and singletons, according to the gene family clustering. The clusters were named with ‘CL’ as the prefix, followed by an ID number, whereas singletons were prefixed with ‘unigene’. Additionally, the similarity among unigenes from the same cluster was greater than 70%.

### Function annotation and classification

The homology searches between the assembled unigenes and the NCBI non-redundant (Nr) database, Swiss-Prot protein database, Kyoto Encyclopedia of Genes and Genomes (KEGG) database and Clusters of Orthologous Groups (COG) database were conducted using the BLASTx algorithm with an E-value of less than 10^–5^, and the best alignments were used to identify the sequence direction. A priority order of Nr, Swiss-Prot, KEGG and COG was followed when the results of different databases conflicted. If the unigenes were unaligned to any of the four public databases, then the software ESTScan [71] was introduced to determine the sequence direction.

Based on the Nr results, the GO annotation of all of the unigenes was performed via the Blast2GO [72] program. Subsequently, WEGO [73] software was used to produce a GO functional classification at the macro level. To annotate and classify the possible functions, the unigenes were also aligned to the COG database. Pathway assignments were performed according to the KEGG pathway database.

### Marker development and Primer design

A total of 57,212 *de novo* sequences were examined to mine for SNP and SSR loci. SSR loci were detected using MicroSAtellite (MISA; http://pgrc.ipk-gatersleben.de/misa/misa.html). In our study, mono-, di-, tri-, tetra-, penta-, and hexa-nucleotides were searched with a minimum of 12, 6, 5, 5, 4, and 4 repeat units, respectively. We first designed 5 primer pairs for each locus using the software Primer 3 and then filtered low-quality primers (S2 Table). Local BLASTn was used to check the redundancy and analyze the chromosome localization with a cut-off of 1.0E-5. The distribution results of the localization on nine chromosomes of *C.sinensis* were depicted using the Mapchart 2.2 software.

SNP identification was performed using the SOAPsnp program (http://soap.genomics.org.cn/soapsnp.html) [74]. SNP primers were designed based on the following criteria via Primer3: primer length of 18–25 bases (optimal 22), PCR product size of 300–600 bp (optimal 400) and annealing temperature of 55–62°C (optimal 58°C).

### Marker amplification and validation

To validate the putative markers, we randomly selected and synthesized 100 SSR primer pairs (S3 Table) and 20 SNP primer pairs (S4 Table). The genomic DNA of ‘Shatian’ pummelo was used as the template for marker validation. The SSR amplification reactions were conducted according to the protocol by Chai et al. [52]. The PCR products were screened using denaturing 6% polyacrylamide gel electrophoresis and stained by ethidium bromide following the protocol of Ruiz et al. [75]. For potential SNP validation, a 20-μl PCR volume was used, containing 1.2 μl of genomic DNA, 0.5 μM each primer, 1×reaction buffer, 200μM each dNTP and 2.5 U FastPfu DNA Polymerase. PCR amplification was performed in a MJ-PTC-200 tm thermal controller (MJ Research, Waltham Mass) using the following steps: 94°C for 10 min, 31 cycles of 94°C for 30 s, 55°C for 30 s, 72°C for 30 s, and a final step at 72°C for 10 min. The amplified products were subjected to 2% agarose gels, and then the products, which were detected using targeted fragments, were sequenced bidirectionally (forward and reverse) in a 10-μl reaction by the Beijing Genomics Institute (BGI, Shenzhen, China).

### SSR polymorphism analysis

The polymorphism on 41 citrus accessions and 3 related species was tested using 29 SSR primers (Table 4) selected from the 82 primers with expected sizes. The PCR amplifications and products detection were performed as previously described. A 0/1 matrixcoding the absence/presence of each band was constructed for further data analysis. The polymorphic information content (PIC), number of alleles, observed heterozygosity (*Ho*) and expected heterozygosity (*He*) were calculated through PowerMarker version 3.25 [76]. A genetic similarity matrix was analyzed using the NTSYSpc 2.1 software [77], and a dendrogram was constructed using the UPGMA method.

## Supporting Information

S1 Fig Length frequency distribution of contigs and unigenes obtained from de novo assembly. (TIF)

S2 Fig Characteristics of similarity search of Shatian pummelo unigenes. (TIF)

S1 Table KEGG classification of Shatian pummelo assembled unigenes. (XLS)

S2 Table The detailed information of all SSR primer pairs for 1174 loci. (XLS)

S3 Table The detailed information of 100 SSR primer pairs. (XLS)

S4 Table The detailed information of 20 SNP primer pairs. (XLS)

S5 Table Validated SNPs using sanger sequencing. (XLS)

## References

[1] Jayaprakasha GK, Girennavar B, Patil BS. Antioxidant capacity of pummelo and navel oranges: Extraction efficiency of solvents in sequence. LWT-Food Science and Technology. 2008; 41: 376–384.

[2] Zhang M, Duan C, Zang Y, Huang Z, Liu G. The flavonoid composition of flavedo and juice from the pummelo cultivar (*Citrus grandis* (L.) Osbeck) and the grapefruit cultivar (*Citrus paradisi*) from China. Food Chemistry. 2011; 129: 1530–1536.

[3] Haruenkit SPR. Investigation of limonoids, flavanones, total polyphenol content and antioxidant activity in seven thai pummelo cultivars. Witthayāsān Kasētsārt: Kasetsart journal Natural sciences Sākhā thammācht. 2009; 43: 458–466. 10.1128/JCM.00852-10 20844212 PMC3020842

[4] Mokbel MS, Hashinaga F. Evaluation of the antioxidant activity of extracts from buntan (*Citrus grandis* Osbeck) fruit tissues. Food Chemistry. 2006; 94: 529–534.

[5] Scora RW. On the history and origin of Citrus. Bulletin of the Torrey Botanical Club. 1975; 102: 369–375.

[6] Yamamoto M, Kubo T, Tominaga S. Self-and cross-incompatibility of various citrus accessions. Journal Japanese Society Horticultural Science. 2006; 75: 372–378.

[7] Fox SE, Geniza M, Hanumappa M, Naithani S, Sullivan C, Preece J, et al De Novo Transcriptome Assembly and Analyses of Gene Expression during Photomorphogenesis in Diploid Wheat *Triticum monococcum* . PloS One. 2014; 9: e96855 10.1371/journal.pone.0096855 24821410 PMC4018402

[8] Shi CY, Yang H, Wei CL, Yu O, Zhang ZZ, Jiang CJ, et al Deep sequencing of the *Camellia sinensis* transcriptome revealed candidate genes for major metabolic pathways of tea-specific compounds. BMC genomics. 2011; 12: 131 10.1186/1471-2164-12-131 21356090 PMC3056800

[9] Emrich SJ, Barbazuk WB, Li L, Schnable PS. Gene discovery and annotation using LCM-454 transcriptome sequencing. Genome research. 2007; 17: 69–73. 17095711 10.1101/gr.5145806 PMC1716268

[10] Iorizzo M, Senalik DA, Grzebelus D, Bowman M, Cavagnaro PF, Matvienko M, et al De novo assembly and characterization of the carrot transcriptome reveals novel genes, new markers, and genetic diversity. BMC Genomics. 2011; 12: 389 10.1186/1471-2164-12-389 21810238 PMC3224100

[11] Gao Z, Luo W, Liu H, Zeng C, Liu X, Yi S, et al Transcriptome analysis and SSR/SNP markers information of the blunt snout bream (*Megalobrama amblycephala*). PLoS One. 2012; 7: e42637 10.1371/journal.pone.0042637 22880060 PMC3412804

[12] Blanca J, Cañizares J, Roig C, Ziarsolo P, Nuez F, Picó B. Transcriptome characterization and high throughput SSRs and SNPs discovery in *Cucurbita pepo* (Cucurbitaceae). BMC genomics. 2011;12: 104 10.1186/1471-2164-12-104 21310031 PMC3049757

[13] Liu Y, Sun Z, Liu D, Wu B, Tao J. Assessment of the genetic diversity of pummelo germplasms using AFLP and SSR markers. Zhongguo nongye kexue. 2004; 38: 2308–2315.

[14] Wu B, Zhong GY, Yue JQ, Yang RT, Li C, Li YJ, et al Identification of Pummelo Cultivars by Using a Panel of 25 Selected SNPs and 12 DNA Segments. PloS One. 2014; 9: e94506 10.1371/journal.pone.0094506 24732455 PMC3986212

[15] Biswas MK, Chai L, Qiang X, Deng X. Generation, functional analysis and utility of *Citrus grandis* EST from a flower-derived cDNA library. Molecular biology reports. 2012; 39: 7221–7235. 10.1007/s11033-012-1553-8 22477149

[16] Reis-Filho JS. Next-generation sequencing. Breast Cancer Res. 2009; 11: S12 10.1186/bcr2431 20030863 PMC2797692

[17] Metzker ML. Sequencing technologies-the next generation. Nature Reviews Genetics. 2009; 11: 31–46. 10.1038/nrg2626 19997069

[18] Feldmeyer B, Wheat CW, Krezdorn N, Rotter B, Pfenninger M. Short read Illumina data for the de novo assembly of a non-model snail species transcriptome (*Radix balthica*, *Basommatophora*, *Pulmonata*), and a comparison of assembler performance. BMC Genomics. 2011; 12: 317 10.1186/1471-2164-12-317 21679424 PMC3128070

[19] Collins LJ, Biggs PJ, Voelckel C, Joly S. An approach to transcriptome analysis of non-model organisms using short-read sequences World Scientific 2008; pp. 3–14. 19425143

[20] Wei W, Qi X, Wang L, Zhang Y, Hua W, Li D, et al Characterization of the sesame (*Sesamum indicum* L.) global transcriptome using Illumina paired-end sequencing and development of EST-SSR markers. BMC genomics. 2011; 12: 451 10.1186/1471-2164-12-451 21929789 PMC3184296

[21] Wang Z, Fang B, Chen J, Zhang X, Luo Z, Huang L, et al De novo assembly and characterization of root transcriptome using Illumina paired-end sequencing and development of cSSR markers in sweetpotato (*Ipomoea batatas*). BMC genomics. 2010; 11: 726 10.1186/1471-2164-11-726 21182800 PMC3016421

[22] Chai L, Ge X, Biswas MK, Xu Q, Deng X. Self-sterility in the mutant ‘Zigui shatian’pummelo (*Citrus grandis* Osbeck) is due to abnormal post-zygotic embryo development and not self-incompatibility. Plant Cell, Tissue and Organ Culture. 2011; 104: 1–11.

[23] Grabherr MG, Haas BJ, Yassour M, Levin JZ, Thompson DA, Amit I, et al Full-length transcriptome assembly from RNA-Seq data without a reference genome. Nature Biotechnology. 2011; 29: 644–652. 10.1038/nbt.1883 21572440 PMC3571712

[24] Kanehisa M, Goto S. KEGG: kyoto encyclopedia of genes and genomes. Nucleic acids research. 2000; 28: 27–30. 10592173 10.1093/nar/28.1.27 PMC102409

[25] Yu K, Xu Q, Da X, Guo F, Ding Y, Deng X. Transcriptome changes during fruit development and ripening of sweet orange (*Citrus sinensis*). BMC genomics. 2012; 13: 10 10.1186/1471-2164-13-10 22230690 PMC3267696

[26] Zenoni S, Ferrarini A, Giacomelli E, Xumerle L, Fasoli M, Malerba G, et al Characterization of transcriptional complexity during berry development in *Vitis vinifera* using RNA-Seq. Plant physiology. 2010; 152: 1787–1795. 10.1104/pp.109.149716 20118272 PMC2850006

[27] Li H, Chen S, Song A, Wang H, Fang W, Guan Z, et al RNA-Seq derived identification of differential transcription in the chrysanthemum leaf following inoculation with *Alternaria tenuissima* . BMC genomics. 2014; 15: 9 10.1186/1471-2164-15-9 24387266 PMC3890596

[28] Su Cl, Chao YT, Alex Chang YC, Chen WC, Chen CY, Lee AY, et al De novo assembly of expressed transcripts and global analysis of the *Phalaenopsis aphrodite* transcriptome. Plant and cell physiology. 2011; 52: 1501–1514. 10.1093/pcp/pcr097 21771864

[29] Roberts A, Pimentel H, Trapnell C, Pachter L. Identification of novel transcripts in annotated genomes using RNA-Seq. Bioinformatics. 2011; 27: 2325–2329. 10.1093/bioinformatics/btr355 21697122

[30] Wu CS, Yu CY, Chuang CY, Hsiao M, Kao CF, Kuo HC, et al Integrative transcriptome sequencing identifies trans-splicing events with important roles in human embryonic stem cell pluripotency. Genome research. 2014; 24: 25–36. 10.1101/gr.159483.113 24131564 PMC3875859

[31] Fu N, Wang Q, Shen HL. De novo assembly, gene annotation and marker development using Illumina paired-end transcriptome sequences in celery (*Apium graveolens* L.). PloS One. 2013; 8: e57686 10.1371/journal.pone.0057686 23469050 PMC3585167

[32] Wang S, Wang X, He Q, Liu X, Xu W, Li L, et al Transcriptome analysis of the roots at early and late seedling stages using Illumina paired-end sequencing and development of EST-SSR markers in radish. Plant cell reports. 2012; 31: 1437–1447. 10.1007/s00299-012-1259-3 22476438

[33] Salgado LR, Koop DM, Pinheiro DG, Rivallan R, Le Guen V, Nicolás MF, et al De novo transcriptome analysis of *Hevea brasiliensis* tissues by RNA-seq and screening for molecular markers. BMC genomics. 2014; 15: 236 10.1186/1471-2164-15-236 24670056 PMC4051172

[34] Cui J, Wang H, Liu S, Zhu L, Qiu X, Jiang Z, et al SNP Discovery from Transcriptome of the Swimbladder of *Takifugu rubripes* . PloS One. 2014; 9: e92502 10.1371/journal.pone.0092502 24651578 PMC3961390

[35] Rosenkranz R, Borodina T, Lehrach H, Himmelbauer H. Characterizing the mouse ES cell transcriptome with Illumina sequencing. Genomics. 2008; 92: 187–194. 10.1016/j.ygeno.2008.05.011 18602984

[36] Gan X, Stegle O, Behr J, Steffen JG, Drewe P, Hildebrand KL, et al Multiple reference genomes and transcriptomes for *Arabidopsis thaliana* . Nature. 2011; 477: 419–423. 10.1038/nature10414 21874022 PMC4856438

[37] Liu F, Sun X, Wang W, Liang Z, Wang F. De novo transcriptome analysis-gained insights into physiological and metabolic characteristics of *Sargassum thunbergii* (Fucales, Phaeophyceae). Journal of Applied Phycology. 2014; 26: 1519–1526.

[38] Varshney RK, Nayak SN, May GD, Jackson SA. Next-generation sequencing technologies and their implications for crop genetics and breeding. Trends in biotechnology. 2009; 27: 522–530. 10.1016/j.tibtech.2009.05.006 19679362

[39] Fullwood MJ, Wei C-L, Liu ET, Ruan Y. Next-generation DNA sequencing of paired-end tags (PET) for transcriptome and genome analyses. Genome research. 2009; 19: 521–532. 10.1101/gr.074906.107 19339662 PMC3807531

[40] Li D, Deng Z, Qin B, Liu X, Men Z. De novo assembly and characterization of bark transcriptome using Illumina sequencing and development of EST-SSR markers in rubber tree (*Hevea brasiliensis* Muell. Arg.). BMC genomics. 2012; 13: 192 10.1186/1471-2164-13-192 22607098 PMC3431226

[41] Barakat A, DiLoreto DS, Zhang Y, Smith C, Baier K, Powell WA, et al Comparison of the transcriptomes of American chestnut (*Castanea dentata*) and Chinese chestnut (*Castanea mollissima*) in response to the chestnut blight infection. BMC Plant Biology. 2009; 9: 51–61. 10.1186/1471-2229-9-51 19426529 PMC2688492

[42] Krost C, Petersen R, Schmidt ER. The transcriptomes of columnar and standard type apple trees (Malus x domestica)- a comparative study. Gene. 2012; 498: 223–230. 10.1016/j.gene.2012.01.078 22353365

[43] Muñoz-Mérida A, González-Plaza JJ, Blanco AM, del Carmen García-López M, Rodríguez JM, Pedrola L, et al De novo assembly and functional annotation of the olive (Olea europaea) transcriptome. DNA research. 2013; 20: 93–108. 10.1093/dnares/dss036 23297299 PMC3576661

[44] Du F, Wu Y, Zhang L, Li XW, Zhao XY, Wang WH, et al. De Novo Assembled Transcriptome Analysis and SSR Marker Development of a Mixture of Six Tissues from Lilium Oriental Hybrid ‘Sorbonne’. Plant Molecular Biology Reporter. 2014; 1–13.

[45] Garg R, Patel RK, Tyagi AK, Jain M. De novo assembly of chickpea transcriptome using short reads for gene discovery and marker identification. DNA research. 2011; 18: 53–63. 10.1093/dnares/dsq028 21217129 PMC3041503

[46] Feng C, Chen M, Xu CJ, Bai L, Yin XR, Li X, et al Transcriptomic analysis of Chinese bayberry (*Myrica rubra*) fruit development and ripening using RNA-Seq. BMC genomics. 2012; 13: 19 10.1186/1471-2164-13-19 22244270 PMC3398333

[47] Wang Y, Pan Y, Liu Z, Zhu X, Zhai L, Xu L, et al De novo transcriptome sequencing of radish (*Raphanus sativus* L.) and analysis of major genes involved in glucosinolate metabolism. BMC genomics. 2013; 14: 836 10.1186/1471-2164-14-836 24279309 PMC4046679

[48] Xu Q, Chen LL, Ruan X, Chen D, Zhu A, Chen C, et al The draft genome of sweet orange (*Citrus sinensis*). Nature genetics. 2013; 45: 59–66. 10.1038/ng.2472 23179022

[49] Liang C, Liu X, Yiu S-M, Lim BL. De novo assembly and characterization of *Camelina sativa* transcriptome by paired-end sequencing. BMC genomics. 2013; 14: 146 10.1186/1471-2164-14-146 23496985 PMC3635884

[50] Sassa H, Kakui H, Minamikawa M. Pollen-expressed F-box gene family and mechanism of S-RNase-based gametophytic self-incompatibility (GSI) in Rosaceae. Sexual plant reproduction. 2010; 23: 39–43. 10.1007/s00497-009-0111-6 20165962

[51] Kao TH, Tsukamoto T. The molecular and genetic bases of S-RNase-based self-incompatibility. The Plant Cell. 2004; 16: S72–S83. 15010517 10.1105/tpc.016154 PMC2643390

[52] Chai L, Biswas MK, Yi H, Guo W, Deng X. Transferability, polymorphism and effectiveness for genetic mapping of the Pummelo (*Citrus grandis* Osbeck) EST-SSR markers. Scientia Horticulturae. 2013; 155: 85–91.

[53] Chen C, Zhou P, Choi YA, Huang S, Gmitter FG JR. Mining and characterizing microsatellites from citrus ESTs. Theoretical and Applied Genetics. 2006; 112: 1248–1257. 16474971 10.1007/s00122-006-0226-1

[54] Peng J, Lapitan NL. Characterization of EST-derived microsatellites in the wheat genome and development of eSSR markers. Functional & integrative genomics. 2005; 5: 80–96. 15650880 10.1007/s10142-004-0128-8

[55] Nuñez-Acuña G, Valenzuela-Muñoz V, Gallardo-Escárate C. High-throughput SNP discovery and transcriptome expression profiles from the salmon louse *Caligus rogercresseyi* (Copepoda: Caligidae). Comparative Biochemistry and Physiology Part D: Genomics and Proteomics. 2014; 10: 9–21. 10.1016/j.cbd.2014.01.003 24561831

[56] Yates SA, Swain MT, Hegarty MJ, Chernukin I, Lowe M, Allison GG, et al De novo assembly of red clover transcriptome based on RNA-Seq data provides insight into drought response, gene discovery and marker identification. BMC genomics. 2014; 15: 453 10.1186/1471-2164-15-453 24912738 PMC4144119

[57] Zhang J, Liang S, Duan J, Wang J, Chen S, Cheng Z, et al De novo assembly and Characterisation of the Transcriptome during seed development, and generation of genic-SSR markers in Peanut (*Arachis hypogaea* L.). BMC genomics. 2012; 13: 90 10.1186/1471-2164-13-90 22409576 PMC3350410

[58] Liu Z, Chen T, Ma L, Zhao Z, Zhao PX, Nan Z, et al Global transcriptome sequencing using the Illumina platform and the development of EST-SSR markers in autotetraploid alfalfa. PloS One. 2013; 8: e83549 10.1371/journal.pone.0083549 24349529 PMC3861513

[59] Dong S, Liu Y, Niu J, Ning Y, Lin S, Zhang Z. De novo transcriptome analysis of the Siberian apricot (*Prunus sibirica* L.) and search for potential SSR markers by 454 pyrosequencing. Gene. 2014; 544: 220–227. 10.1016/j.gene.2014.04.031 24746601

[60] Yu Y, Wei J, Zhang X, Liu J, Liu C, Li F, et al SNP Discovery in the Transcriptome of White Pacific Shrimp *Litopenaeus vannamei* by Next Generation Sequencing. PloS One. 2014; 9: e87218 10.1371/journal.pone.0087218 24498047 PMC3907553

[61] Barrett H, Rhodes A. A numerical taxonomic study of affinity relationships in cultivated Citrus and its close relatives. Systematic Botany. 1976; 1: 105–136.

[62] Nicolosi E, Deng Z, Gentile A, La Malfa S, Continella G, Tribulato E. Citrus phylogeny and genetic origin of important species as investigated by molecular markers. Theoretical and Applied Genetics. 2000; 100: 1155–1166.

[63] García-Lor A, Luro F, Navarro L, Ollitrault P. Comparative use of InDel and SSR markers in deciphering the interspecific structure of cultivated citrus genetic diversity: a perspective for genetic association studies. Molecular genetics and genomics. 2012; 287: 77–94. 10.1007/s00438-011-0658-4 22160318

[64] Garcia-Lor A, Curk F, Snoussi-Trifa H, Morillon R, Ancillo G, Luro F, et al A nuclear phylogenetic analysis: SNPs, indels and SSRs deliver new insights into the relationships in the ‘true citrus fruit trees’ group (Citrinae, Rutaceae) and the origin of cultivated species. Annals of botany. 2013; 111: 1–19. 10.1093/aob/mcs227 23104641 PMC3523644

[65] Ollitrault P, Terol J, Garcia-Lor A, Bérard A, Chauveau A, Froelicher Y, et al SNP mining in *C*. *clementina* BAC end sequences; transferability in the *Citrus genus* (Rutaceae), phylogenetic inferences and perspectives for genetic mapping. BMC genomics. 2012; 13: 13 10.1186/1471-2164-13-13 22233093 PMC3320530

[66] Luro F, Gatto J, Costantino G, Pailly O. Analysis of genetic diversity in *Citrus* . Plant Genetic Resources. 2011; 9: 218–221.

[67] Hamon P, Seguin M, Perrier X, Glaszmann JC. Genetic diversity of cultivated tropical plants Science, USA, 2003.

[68] Fanciullino A-L, Dhuique-Mayer C, Luro F, Morillon R, Ollitrault P. Carotenoid biosynthetic pathway in the *Citrus genus*: number of copies and phylogenetic diversity of seven genes. Journal of Agricultural and Food Chemistry. 2007; 55: 7405–7417. 17691802 10.1021/jf070711h

[69] Liu YZ, Liu Q, Tao N-G, Deng XX. Efficient isolation of RNA from fruit peel and pulp of ripening navel orange(*Citrus sinensis Osbeck*). J Huazhong Agric Univ. 2006; 25: 300–304.

[70] Cheng YJ, Guo WW, Yi HL, Pang XM, Deng XX. An efficient protocol for genomic DNA extraction fromCitrus species. Plant Molecular Biology Reporter. 2003; 21: 177–178.

[71] Iseli C, Jongeneel CV, Bucher P. ESTScan: a program for detecting, evaluating, and reconstructing potential coding regions in EST sequences. Proc Int Conf Intell Syst Mol Biol. 1999; 7:138–148. 10786296

[72] Conesa A, Gotz S, Garcia-Gomez JM, Terol J, Talon M, Robles M. Blast2GO: a universal tool for annotation, visualization and analysis in functional genomics research. Bioinformatics. 2005; 21: 3674–3676. 16081474 10.1093/bioinformatics/bti610

[73] Ye J, Fang L, Zheng HK, Zhang Y, Chen J, Zhang Z, et al WEGO: a web tool for plotting GO annotations. Nucleic Acids Research. 2006; 34: W293–W297. 16845012 10.1093/nar/gkl031 PMC1538768

[74] Li R, Yu C, Li Y, Lam TW, Yiu SM, Kristiansen K, et al SOAP2: an improved ultrafast tool for short read alignment. Bioinformatics. 2009; 25: 1966–1967. 10.1093/bioinformatics/btp336 19497933

[75] Ruiz C, Breto MP, Asins M. A quick methodology to identify sexual seedlings in citrus breeding programs using SSR markers. Euphytica. 2000; 112: 89–94.

[76] Liu K, Muse SV. PowerMarker: an integrated analysis environment for genetic marker analysis. Bioinformatics. 2005; 21: 2128–2129. 15705655 10.1093/bioinformatics/bti282

[77] Rohlf FJ. NTSYS-pc Numerical taxonomy and multivariate analysis system.Version 2.00. Exeter Software Setauket, New York 1998

