# Supplementary figures and images for: De Novo Transcriptome Assembly of Pummelo and Molecular Marker Development
Source: PLoS One. 2015 Mar 23;10(3):e0120615. doi: 10.1371/journal.pone.0120615 (PMC4370633; doi:10.1371/journal.pone.0120615)

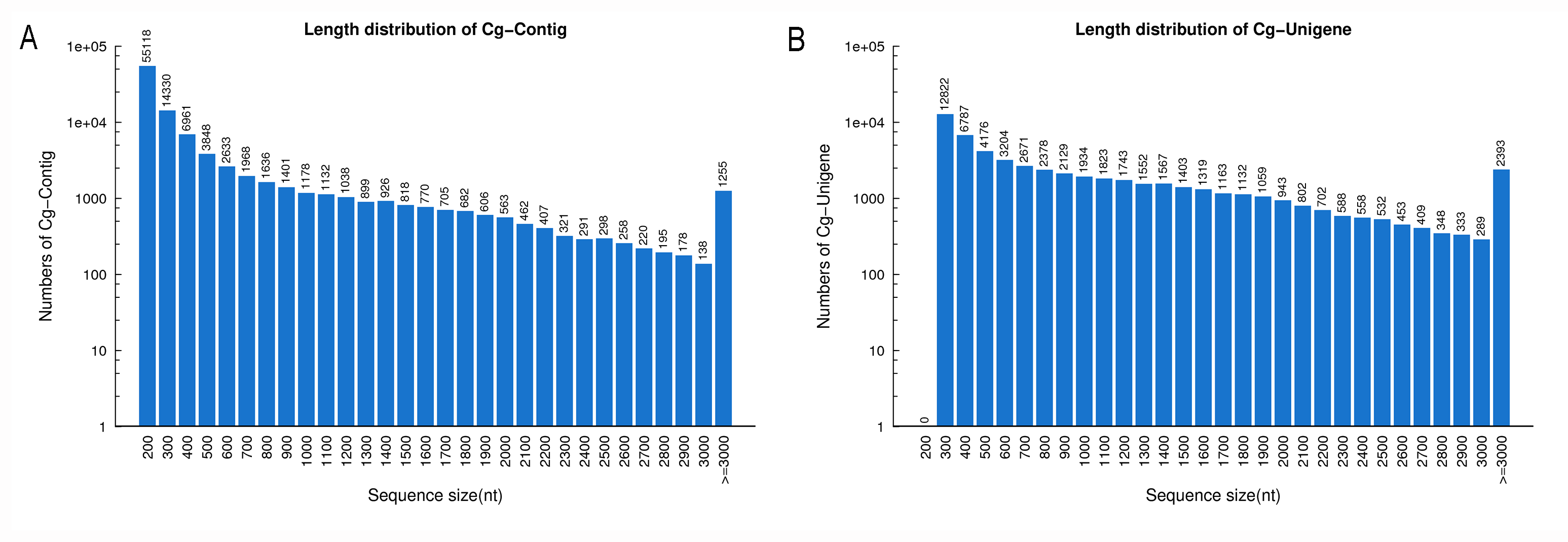

Supplement: S1 Fig — (TIF) [file pone.0120615.s001.tif]

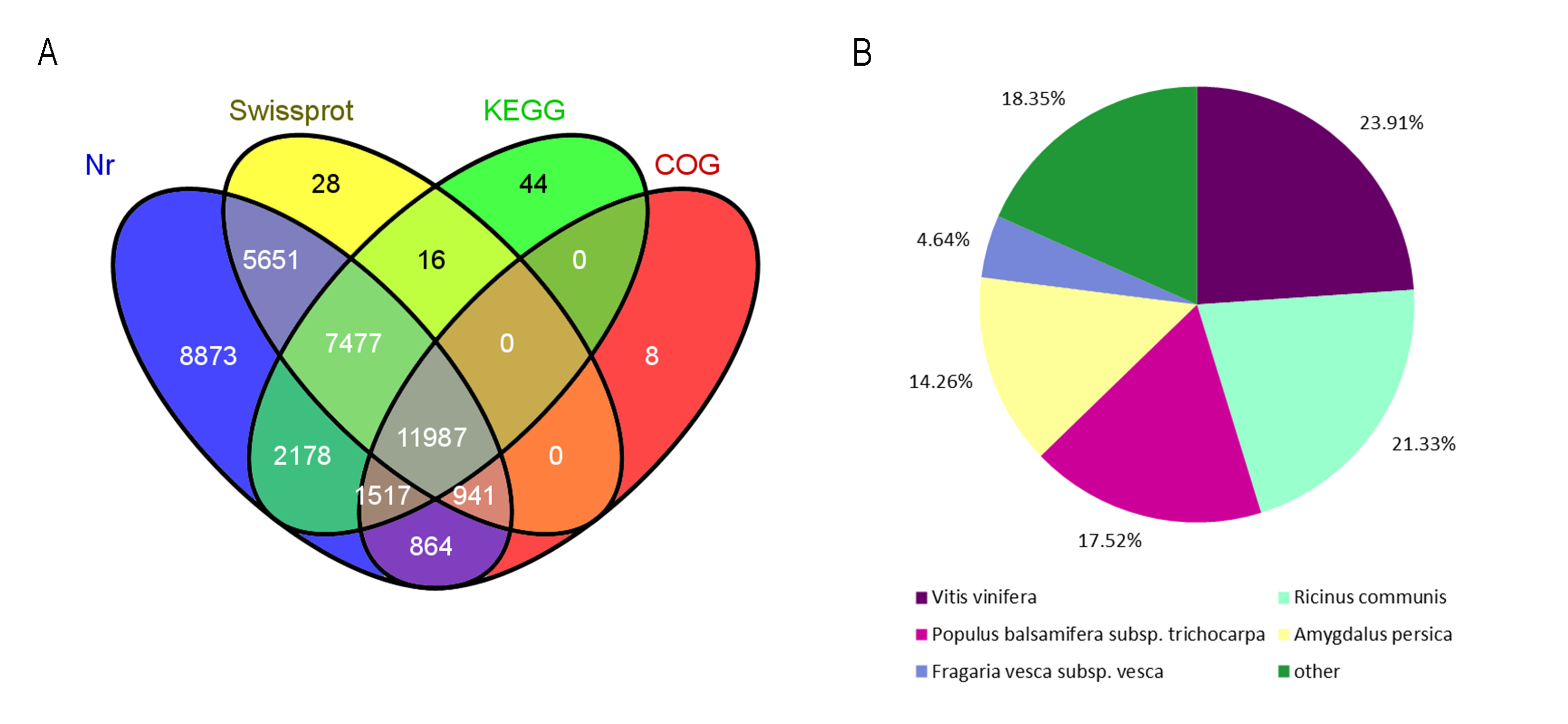

Supplement: S2 Fig — (TIF) [file pone.0120615.s002.tif]
